# Mosquito (Diptera: Culicidae) populations in contrasting areas of the western regions of Burkina Faso: species diversity, abundance and their implications for pathogen transmission

**DOI:** 10.1186/s13071-023-06050-2

**Published:** 2023-11-27

**Authors:** Didier P. Alexandre Kaboré, Dieudonné Diloma Soma, Patricia Gil, Mahamadi Kientega, Simon P. Sawadogo, Georges Anicet Ouédraogo, Philippe Van de Perre, Thierry Baldet, Serafin Gutierrez, Roch K. Dabiré

**Affiliations:** 1 Institut de Recherche en Sciences de la Santé (IRSS), 01BP 545 Bobo-Dioulasso, Burkina Faso; 2 Université Nazi BONI, Bobo-Dioulasso, Burkina Faso; 3 ASTRE Research Unit, CIRAD, INRAe, Montpellier University, Montpellier, France; 4 Pathogenesis and Control of Chronic and Emerging Infections, INSERM, University of Montpellier, EFS; CHU Montpellier, Montpellier, France

**Keywords:** Mosquitoes, Environment type, Diversity, Species richness, Burkina Faso

## Abstract

**Background:**

Mosquitoes (Diptera: Culicidae) can have a significant negative impact on human health. The urbanization of natural environments and their conversion for agricultural use, as well as human population growth, may affect mosquito populations and increase the risk of emerging or re-emerging mosquito-borne diseases. We report on the variety and number of adult mosquitoes found in four environments with varying degrees of human impact (rural, urban, rice fields, and forest) located in a savannah zone of West Africa.

**Methods:**

Mosquitoes were collected from two regions (Hauts-Bassins and Sud-Ouest) of Burkina Faso during five periods between August 2019 and June 2021. Sampling sites were grouped according to environment. Mosquitoes were collected using BG-Sentinel traps and double net traps, and Prokopack Aspirators. Statistical analyses were performed using R software version 4.1.2. Logistic regression, using generalised mixed linear models, was used to test the effect of environment on mosquito abundance and diversity. Alpha diversity analysis was also performed, using the vegan package.

**Results:**

A total of 10,625 adult mosquitoes were collected, belonging to 33 species and five genera: *Culex*, *Aedes*, *Anopheles*, *Mansonia*, and *Ficalbia*. The most dominant species were *Culex quinquefasciatus*, *Anopheles gambiae* sensu lato and *Aedes aegypti*. Alpha diversity was similar in the two regions. Habitat had a significant effect on mosquito species richness, the Shannon index and the Simpson index. The rural environment had the highest species richness (*n* = 28) followed by the forest environment (*n* = 24). The highest number of mosquitoes (4977/10,625) was collected in the urban environment.

**Conclusions:**

The species composition of the mosquito populations depended on the type of environment, with fewer species in environments with a high human impact such as urban areas and rice fields. Due to the diversity and abundance of the mosquito vectors, the human populations of all of the environments examined are considered to be at potential risk of mosquito-borne diseases.

**Graphical Abstract:**

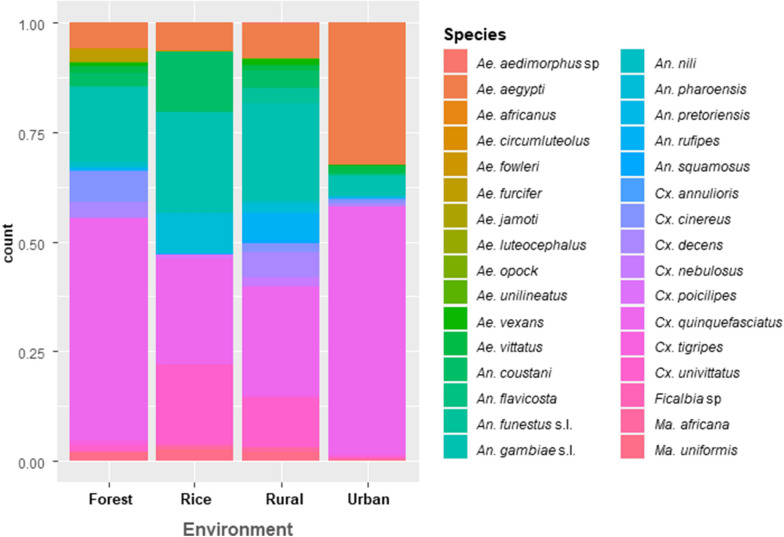

## Background

The urbanisation of the natural environment and its modification due to agricultural practices and population growth expose human communities to new ecological constraints as a consequence of (i) the extinction of certain animal and plant species; (ii) variations in climatic conditions (global warming, drought, extreme climatic events); and (iii) the risk of the emergence of viral and/or parasitic diseases through disruption of the natural cycles involving hosts and pathogens. In Burkina Faso, for example, cases of cutaneous leishmaniasis were recorded in the capital, Ouagadougou, between 2000 and 2005, probably due to the urbanisation of a peripheral district of the city which favoured contact between the rodent reservoir, the sandfly vector and the urban human population [1, 2]. Bonds et al. [3] reported that biodiversity loss is a major factor in the spread of vector-borne diseases (e.g. dengue, malaria, leishmaniasis), which in turn have negative impacts on the economy and human health. Changes in land use can alter the diversity, distribution, abundance and feeding patterns of mosquito populations due to alterations in the landscapes they occupy [4]. Increasing international travel and globalization also favour the geographical spread of mosquito species, which may thus modify the Culicidae community in the areas that they invade. For example, the mosquitoes *Aedes albopictus*, *Aedes japonicus* and *Aedes koreicus*, which are endemic to Asia, have colonised several European countries [5, 6]. Both *Ae. aegypti* and *Ae. albopictus* have been recently found on the island of Cyprus [7]. The recent invasion of East Africa by *Anopheles stephensi*, a malaria vector of urban areas in India, is extremely worrying as it could jeopardise current malaria control efforts [8].

Changes in mosquito populations can influence the transmission dynamics of emerging and re-emerging infectious diseases that are transmitted by them [4, 9]. For example, anthropogenic modifications can have a positive effect on mosquito vector populations by creating favourable breeding conditions for them [10, 11]. In recent years, studies of medically important insects such as mosquitoes have been focused on gaining a more accurate understanding of the ecology of these vectors and the interactions between them and/or the pathogens that they transmit. The aim of many of these studies was to develop effective strategies to interrupt the transmission of vector-borne diseases. However, studies describing mosquito populations in the field are scarce. Understanding the abundance and spatial distribution of mosquitoes in different landscapes subject to multiple climatic and anthropogenic disturbances is essential for assessing the risk of vector-borne disease transmission. Our study was undertaken to update information on the diversity and abundance of mosquito populations in different environments (urban, rural, rice fields and forest) characteristic of a West African savannah region.

## Methods

### Study sites

**Fig. 1:**
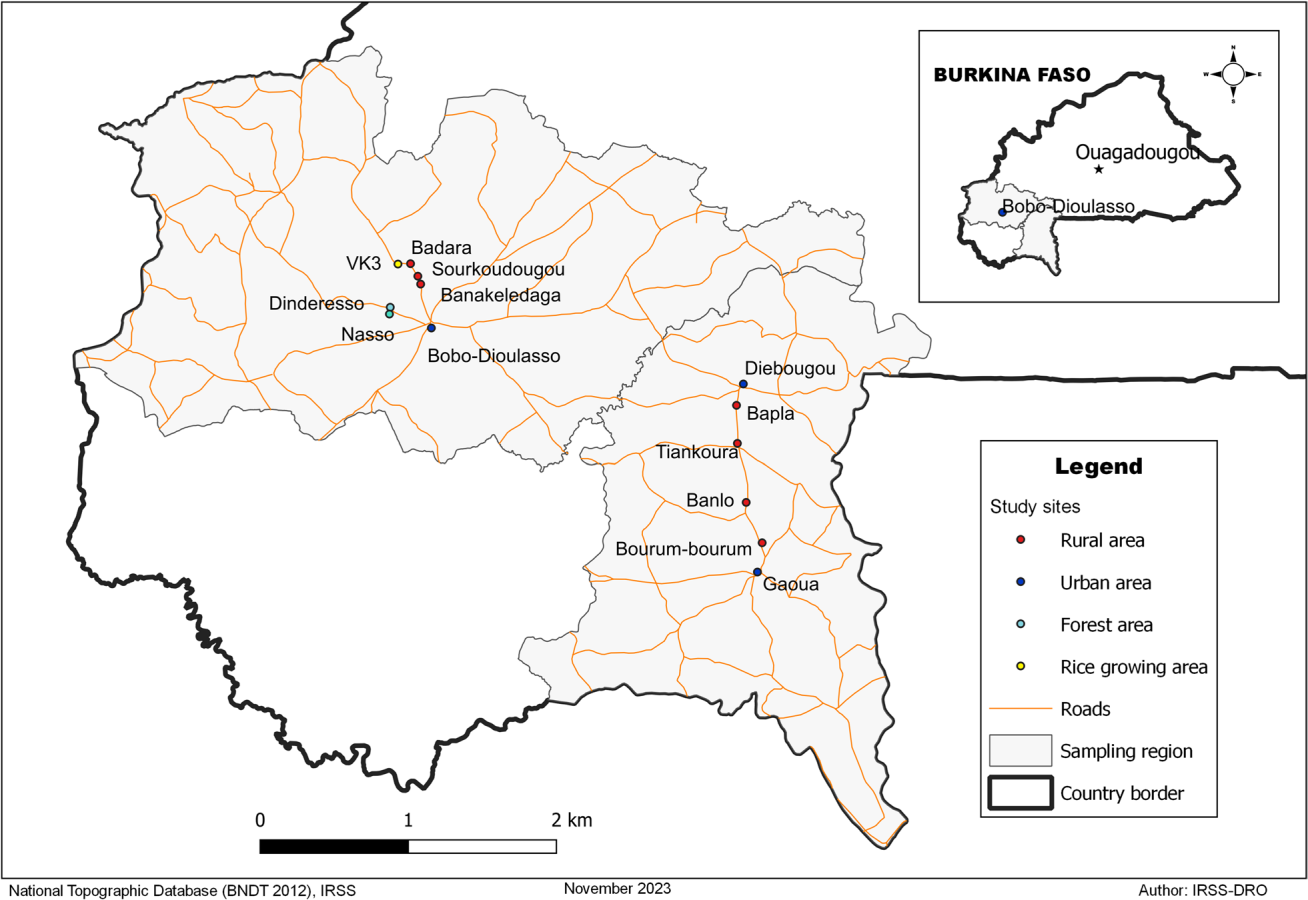
Location of mosquito sampling sites in Burkina Faso. Each dot represents one sampling site. Samples were grouped according to type of environment, as follows: rural, urban, rice fields and forest

The mosquitoes were collected in the Hauts-Bassins and Sud-Ouest regions of Burkina Faso. These regions are affected by urbanisation, the increased use of land for agriculture and the development of artisanal gold mining (Fig. 1). They have an average annual rainfall of 1200 mm. The climate is tropical with two seasons: a rainy season from June to September and a dry season from October to May. The vegetation of the Hauts-Bassins region is mainly composed of tree savannah (lower total plant density) and that of the Sud-Ouest region wooded savannah (higher total plant density). Agriculture is the main economic activity in both regions, followed by artisanal gold mining in the Sud-Ouest region. 

In the Hauts-Bassins region, the sampling sites were located along two road transects. Sampling started in the town of Bobo-Dioulasso. On the first transect, samples were collected in three rural areas (Banakeledaga, Sourkoudougou, Badara) and in the Vallée du Kou 3 (VK3), the rice-growing area. The ecosystem in these locations is wooded savannah on low-lying land with a very flat topography. The main crops are cereals (mainly rice in VK3) and banana and papaya. Housing mainly comprises traditional or semi-modern houses. Sampling on the second transect was carried out in two forest areas, Nasso and Dinderesso, where the dwellings are semi-modern, and agriculture is essentially cereal based. 

In the Sud-Ouest region, sampling was carried out on a single road transect located between two urban areas: Diébougou and Gaoua. In addition to the two urban areas, sampling was carried out in four rural sites characterized by wooded savannah: Bapla, Tiankoura, Banlo and Bouroum-Bouroum.

### Mosquito sampling and identification

Mosquito sampling was conducted during five periods (August and September 2019, June and July 2020, October 2020, May 2021, and June 2021) to cover the different climatic seasons in the region to achieve the best representativeness and optimise the sampling in terms of abundance and species richness. Sampling was carried out on 2 consecutive days. In each locality, a house was chosen for the sampling of mosquitoes outside. Three types of devices were used: a double net trap [12], a BG-Sentinel trap (Biogents, Germany) baited with BG-Lure (Biogents) and CO_2_ (BG trap) [13], and a Prokopack Aspirator [14]. Two double net traps were set up at two houses at least 100 m apart, one with a human volunteer and the other one with an animal (cattle) as the bait to attract mosquitoes. The human volunteer and the animal were protected by the first net, which prevented the mosquitoes from biting them. These traps were used between 6 p.m. and 7 a.m. Five BG traps were set up in five houses at 8 a.m. and operated continuously over the 2 sampling days. A Prokopack Aspirator was used to collect mosquitoes in 30 resting places (agricultural huts, abandoned houses, livestock pens and abandoned tires) around these dwellings from 6 a.m. to 11 a.m. on the 2 sampling days. The specimens were identified morphologically using identification keys [15–17], grouped according to date, site and environment and stored at − 80 °C for subsequent analyses.

### Statistical analysis

Statistical analyses were performed with R version 4.1.2. A Kruskal–Wallis test was used to compare mean abundance between environment types. Logistic regression by generalized mixed linear models was used to test the effect of environment on mosquito abundance. Species richness and Shannon and Simpson diversity indices for the two regions and four collection environments were calculated using the vegan package [18]. Logistic regression by generalized linear models was used to test the effect of environment on each diversity index, and the emmeans package was used to compare the indices between environments.

## Results

### Mosquito abundance and species composition

A total of 10,625 mosquitoes were collected over the five sampling periods at 13 sites distributed across the four environments. The total number of mosquitoes was significantly different between environments (Kruskal–Wallis, χ^*2*^ = 22.29,* df* = 3, *P* < 0.001). As expected, a greater number of female mosquitoes were collected (7221/10,625, corresponding to 67.96%). Thirty-three species were identified, which belonged to the following genera: *Culex*, *Aedes*, *Anopheles*, *Mansonia*, and *Ficalbia*. Based on the total number of mosquitoes collected per sampling method, the Prokopack Aspirator was the most effective, followed by the BG trap, the double net plus animal trap and the double net plus human trap (Table 1). The highest diversity of mosquitoes (29 out of 33 species) was collected by the double net plus animal trap followed by the double net plus human trap (26 out of 33 species), the Prokopack Aspirator (19 out of 33 species) and the BG trap (17 out of 33 species) (Table 1). More species of *Anopheles* were collected in the double net traps, but the Prokopack Aspirator and the BG trap were more specific for the sampling of *Ae. aegypti* and *Culex quinquefasciatus* (Table 1). The distribution of culicids varied with environment and sampling period. Culicids were more abundant in urban areas (46.84%) and rural areas (29.28%) (Table 2). Among the mosquito genera, *Culex* predominated (53.92% of culicids) followed by *Anopheles* (23.7%) and *Aedes* (21.14%) (Table 2). Nine species of *Anopheles* were collected, predominantly from rural and rice field environments, at 39.24% and 46.22%, respectively (Table 2). Species of *Aedes* were most abundant in urban areas (34.04%). They represented 10.92% of the collected mosquitoes in rural areas and 11.41% in the forest environment. A total of 12 *Aedes* species were collected during this study, with *Ae. aegypti* predominating and accounting for 89.72% of the collected *Aedes* mosquitoes followed by *Aedes vittatus* at 5.65%. Nine species of *Culex* were identified, with *Cx. quinquefasciatus* being the predominant one at 78.22%. These species were predominant in the urban area at 59.41% and in the rural area at 46.90%. Other culicids, such as *Aedes furcifer*, *Aedes fowleri*, *Aedes jamoti*, *Anopheles pretoriensis*, *Culex nebulosus* and *Culex uniformis*, were observed in low numbers (Table 2).

**Table 1:** Mosquito species abundance by sampling method

Genus	Species	Prokopack	BG trap	Tent trap + animal	Tent trap + human	Relative abundance (%)
*Aedes*	*Aedes aedimorphus sp.*	0	0	1	0	0
	*Aedes aegypti*	1650	321	27	18	18.97
	*Aedes africanus*	1	0	0	0	0.01
	*Aedes circumluteolus*	0	0	2	0	0.02
	*Aedes fowleri*	0	1	5	0	0.06
	*Aedes furcifer*	0	0	16	8	0.23
	*Aedes jamoti*	0	0	2	1	0.03
	*Aedes luteocephalus*	0	0	0	1	0.01
	*Aedes opock*	0	0	0	3	0.03
	*Aedes unilineatus*	0	1	2	0	0.03
	*Aedes vexans*	4	0	36	20	0.56
	*Aedes vittatus*	5	5	48	69	1.20
*Anopheles*	*Anopheles coustani*	16	2	311	60	3.66
	*Anopheles flavicosta*	5	0	8	2	0.14
	*Anopheles funestus* sensu lato (s.l.)	39	15	46	10	1.04
	*Anopheles gambiae* s.l.	398	451	281	297	13.43
	*Anopheles nili*	14	1	24	22	0.57
	*Anopheles pharoensis*	9	0	158	45	2.00
	*Anopheles pretoriensis*	0	0	3	0	0.03
	*Anopheles rufipes*	11	18	178	4	1.99
	*Anopheles squamosus*	0	0	16	8	0.23
*Culex*	*Culex annulioris*	0	0	1	7	0.08
	*Culex cinereus*	4	79	63	38	1.73
	*Culex decens*	174	20	37	22	2.38
	*Culex nebulosus*	0	0	67	2	0.65
	*Culex poicilipes*	2	1	6	12	0.20
	*Culex quinquefasciatus*	2115	1539	591	239	42.20
	*Culex tigripes*	7	3	10	14	0.32
	*Culex uniformis*	0	0	20	0	0.19
	*Culex univittatus*	114	54	392	97	6.18
*Mansonia*	*Mansonia africana*	5	2	30	29	0.62
	*Mansonia uniformis*	20	9	57	43	1.21
*Ficalbia*	*Ficalbia* sp.	0	0	0	1	0.01
Total		4593	2522	2438	1072	10625
Total species		19	17	29	26	33

**Table 2:** Species composition of the mosquito population in each environment

		Forest	Rice	Rural	Urban	Total
Genus	Species	% (*n*)	% (*n*)	% (*n*)	% (*n*)	
*Aedes*	*Aedes aedimorphus sp.*	0	0	0.032 (1)	0	0 (1)
	*Aedes aegypti*	5.70 (57)	6.37 (98)	7.97 (248)	32.40 (1613)	19 (2016)
	*Aedes africanus*	0	0	0.032 (1)	0	0 (1)
	*Aedes circumluteolus*	0.10 (1)	0.065 (1)	0	0	0.01 (2)
	*Aedes fowleri*	0.60 (6)	0	0	0	0.05 (6)
	*Aedes furcifer*	2.402 (24)	0	0	0	0.22 (24)
	*Aedes jamoti*	0	0	0.096 (3)	0	0.02 (3)
	*Aedes luteocephalus*	0.10 (1)	0	0	0	0 (1)
	*Aedes opock*	0.10 (1)	0	0.064 (2)	0	0.2 (3)
	*Aedes unilineatus*	0.10 (1)	0	0.064 (2)	0	0.2 (3)
	*Aedes vexans*	0.70 (7)	0	1.285 (40)	0.26 (13)	0.56 (60)
	*Aedes vittatus*	1.60 (16)	0	1.382 (43)	1.36 (68)	0.2 (127)
	Total *Aedes*	11.41 (114)	6.43 (99)	10.92 (340)	34.3 (1694)	2247
*Anopheles*	*Anopheles coustani*	3.00 (30)	13.784 (212)	3.66 (114)	0.66 (33)	3.66 (389)
	*Anopheles flavicosta*	0	0	0.48 (15)	0	0.14 (15)
	*Anopheles funestus* s.l.	0.30 (3)	0	3.182 (99)	0.16 (8)	1.03 (110)
	*Anopheles gambiae* s.l.	16.81 (168)	23.14 (356)	22.14 (689)	4.29 (214)	13.43 (1427)
	*Anopheles nili*	1.00 (10)	0	0.80 (25)	0.52 (26)	0.57 (61)
	*Anopheles pharoensis*	0.50 (5)	9.037 (139)	1.99 (62)	0.12 (6)	2 (212)
	*Anopheles pretoriensis*	0	0	0.096 (3)	0	0.02 (3)
	*Anopheles rufipes*	0.60 (6)	0	6.23 (194)	0.22 (11)	2 (211)
	*Anopheles squamosus*	0	0.260 (4)	0.642 (20)	0	0.22 (24)
	Total *Anopheles*	22.22 (222)	46.22 (711)	29.24 (1221)	5.98 (298)	2452
*Culex*	*Culex annulioris*	0.20 (2)	0	0	0.12 (6)	0.07 (8)
	*Culex cinereus*	7.00 (7)	0	2.05 (64)	1 (50)	1.73 (184)
	*Culex decens*	3.60 (36)	0.195 (3)	5.785 (180)	0.68 (34)	2.38 (253)
	*Culex nebulosus*	0	0	2.217 (69)	0	0.64 (69)
	*Culex poicilipes*	0.10 (1)	0.975 (15)	0.128 (4)	0.02 (1)	0.19 (21)
	*Culex quinquefasciatus*	50.85 (508)	24.12 (371)	24.943 (776)	56.84 (2829)	42.2 (4484)
	*Culex tigripes*	1.00 (10)	0.065 (1)	0.38 (12)	0.22 (11)	0.32 (34)
	*Culex uniformis*	0	0	0.64 (20)	0	0.18 (20)
	*Culex univittatus*	1.50 (15)	18.33 (282)	10.73 (334)	0.52 (26)	6.18 (657)
	Total *Culex*	64.26 (642)	43.69 (672)	46.89 (1459)	59.41 (2957)	5730
*Mansonia*	*Mansonia africana*	0.10 (1)	1.170 (18)	0.835 (26)	0.42 (21)	0.62 (66)
	*Mansonia uniformis*	2.00 (20)	2.405 (37)	2.089 (65)	0.14 (7)	1.21 (129)
	Total *Mansonia*	2.1 (21)	3.57 (55)	2.92 (91)	0.56 (28)	195
*Ficalbia*	*Ficalbia* sp	0	0.0650 (1)	0	0	0 (1)
Total		9.40 (999)	14.47 (1538)	29.28 (3111)	46.84 (4977)	10625

### Alpha diversity

**Fig. 2:**
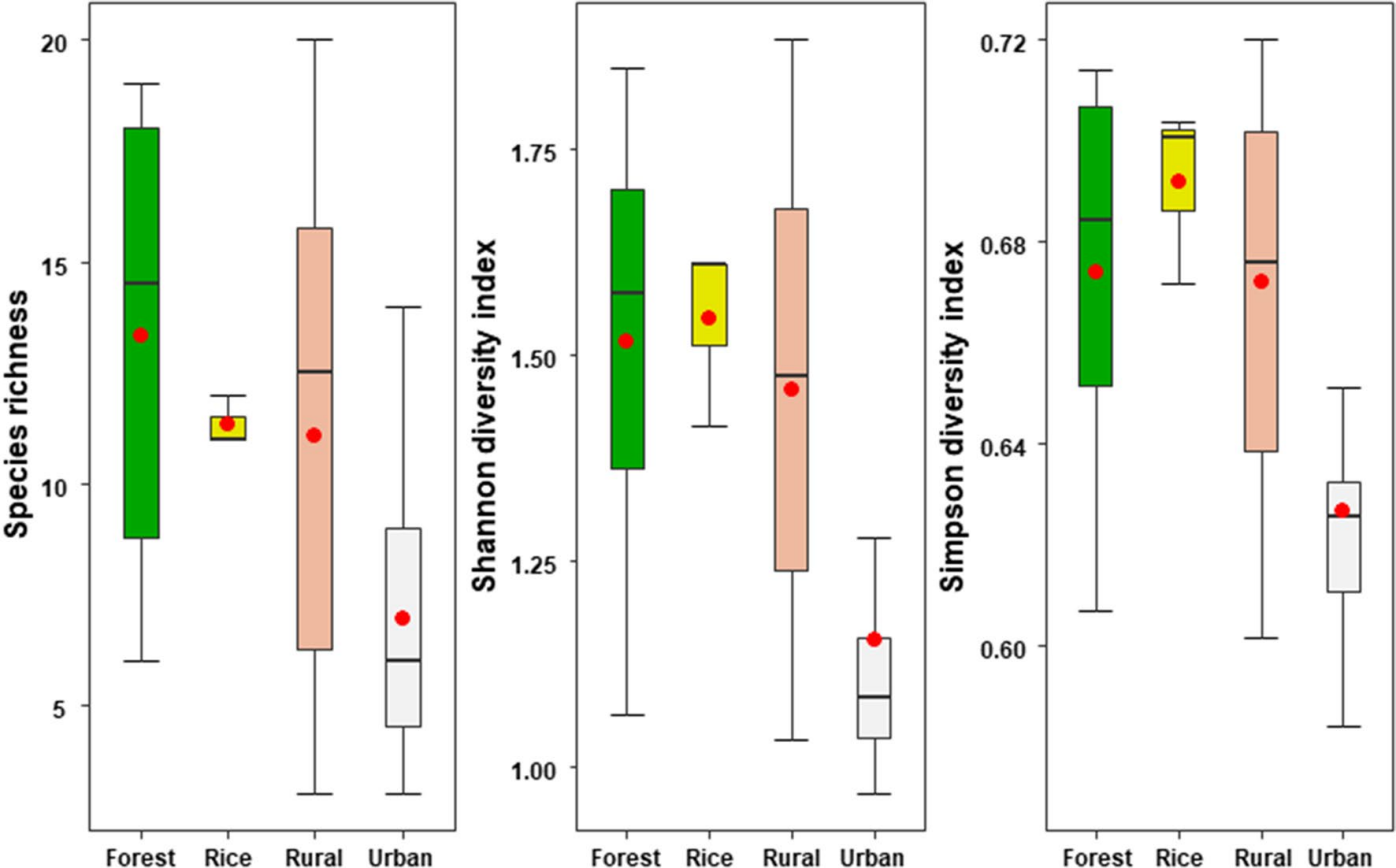
Alpha diversity indices for mosquitoes collected in each environment

No significant differences in species richness between the Sud-Ouest and Hauts-Bassins regions were shown by alpha diversity (*P* > 0.05) or Shannon (*P* > 0.05) and Simpson (*P* > 0.05) indices. Environment had a significant effect on mosquito species richness [likelihood ratio test (LRT), χ^2^ = 14.79, *df* = 3, *P* < 0.001), and Shannon (LRT, χ^2^ = 26.57, *df* = 3, *P* < 0.001) and Simpson (LRT, χ^2^ = 23.59, *df* = 3, *P* < 0.001)] indices. Significant differences in species richness (emmeans, *Z* = 3.41; *SE* = 0.44; *P* < 0.05), and diversity [Shannon (emmeans, *Z* = 3.92; *SE* = 0.26; *P* < 0.05) and Simpson (emmeans, *Z* = 3.24; *SE* = 0.33; *P* < 0.05) indices] were observed between the forest and urban environments (Fig. 2). Species richness differed slightly between the urban and rice field environments (emmeans *Z* = 1.95; *SE* = 0.49; *P* = 0.05). In contrast, the Shannon (emmeans, *Z* = 3.34; SE = 0.34; *P* < 0.05) and Simpson (emmeans, *Z* = 3.44; SE = 0.18; *P* < 0.05) indices showed the highest diversity of culicids in the rice field environment compared to the urban environment, where it was lowest (Fig. 2). The urban environment was less diverse than the rural environment, as shown by species richness (emmeans *Z* = 3.02; SE = 0.3;* P* < 0.05), and the Shannon (emmeans, *Z* = 4.35; SE = 0.19; *P* < 0.05) and Simpson (emmeans, *Z* = 4.14; SE = 0.1; *P* < 0.05) indices (Fig. 2).

## Discussion

Human activities can modify the natural environment and provide new ecological niches that may drive a mosquito species towards adaptation or extinction. Here, we investigated the relationship between mosquito diversity and habitat modification by humans across a range of sites, comprising rural, urban, rice field and forest areas, in savannah areas of western Burkina Faso.

More mosquitoes were collected with the Prokopack Aspirator than the BG trap and the double net traps (human or animal bait). The efficacy of the Prokopack Aspirator can be explained by the fact that it is an active method that requires a technician with entomological training to search for and collect mosquitoes from potential resting sites [14]. In contrast, the BG and double net traps have been developed to capture host-seeking mosquitoes, i.e. by using a lure and CO_2_ as the attractants with the BG trap and a human or a bovine host for the double net trap. These collection methods are considered passive, as they only collect specimens attracted to the traps, and depend on the attractant used; the trap yields vary greatly in terms of mosquito abundance and diversity, which also depend on the context, such as the climate, environment, and type of habitat [19]. This probably explains the low density of mosquitoes collected with these types of traps during our study. It should also be noted that the types of traps we used preferentially collect female mosquitoes that usually feed on mammals [20]; for the collection of ornithophilic species, other types of traps should be used, such as bird-baited traps [21] or the recently developed nest mosquito trap [22]. In the present study, the double net plus animal trap was more effective than the double net plus human trap. This may have been due to the fact that cattle emit more CO_2_, which is a common host-seeking cue for mosquitoes [23], than humans. The effectiveness of these traps could also be explained by the abundance of zoophilic mosquitoes and the presence of many animals in the different environments sampled.

We collected a total of 10,625 mosquitoes representing five genera and 33 species from the four types of environments. All of the species are common members of the culicid fauna of Burkina Faso. The mosquito abundance varied greatly depending on the type of environment, with the highest abundances found in the urban and the rural sites (Table 2). The mosquito population in the urban environment had the lowest species diversity, consisting of a core community comprising the three most frequent species: *Cx. quinquefasciatus*, *Ae. aegypti* and *An. gambiae*. The predominance and abundance of these species in urban environments can be explained by their co-adaptation to areas in which humans live with respect to the availability of sites suitable for their larvae, trophic preferences and resting places. *Culex quinquefasciatus* is known to use polluted breeding sites. Population growth and urbanization lead to an increase in these potential breeding sites, and consequently the abundance of this species in cities and towns throughout the tropics [24]. In Bobo Dioulasso, Burkina Faso’s second largest city in the southwestern savannah region, *Cx*. *quinquefasciatus* was the main mosquito species identified as early as 1970 biting humans [25]. Other studies carried out in the same town confirmed its predominance and its aggressiveness towards humans [26]. *Anopheles gambiae* sensu lato (s.l.) mosquitoes like unpolluted stagnant water without submerged vegetation for oviposition and larval development [27, 28]. Highly urbanised city centres are not very favourable for *Anopheles* malaria vectors, whereas certain areas along rivers or in low-lying areas are. *Anopheles arabiensis*, a member of the *An. gambiae* s.l. complex and a major vector of malaria, which was originally distributed in the dry Sahelian regions, is now present in many West African towns [29]. In Bobo-Dioulasso, this species has become the dominant malaria vector [30, 31], whereas it was formerly present only at a low abundance [32]. In West Africa, *An. arabiensis* is now found in towns in the more humid areas of the forest belt of the Gulf of Guinea, such as in Nigeria [33] and Côte d'Ivoire [34]. The adaptation of this species to pollution [35] and climate change (rising temperatures, drought) is thought to promote its proliferation in West African cities. Female *Ae. aegypti* mostly lay their eggs in domestic and peridomestic water containers. The availability of these containers is partly due to socioeconomic activities [36, 37]. Females of this species preferentially bite humans and rest inside dwellings, and thus find all the necessary conditions to proliferate in human habitats in urban and rural areas of West Africa [38]. It is important to note the absence from our samples of the related species *Ae. albopictus*, the notorious Asian tiger mosquito, and particularly its absence from the urban sites near Côte d'Ivoire, Ghana and Mali, where this invasive species has recently been detected [39]. In addition, Robert et al. [32] reported certain species, such as *Ae. fowleri*, *Ae. luteocephalus*, *Ae. hirsutus*, in urban environments in Bobo-Dioulasso, which were not found in our study. Their absence could be due to increasing urbanization over the four last decades, which may be unfavourable for these species, which develop in natural breeding sites.

Previous studies carried out in the same western part of Burkina Faso reported the predominance of these three species in urban areas, with *Cx*. *quinquefasciatus* always being the most abundant culicid species, followed by *An. gambiae* s.l. and *Ae. aegypti* [31, 32]. A core community comprising these three species is generally found in most towns in West Africa and also in East Africa [40]. These three species can expose urban human populations to several parasitic and arboviral diseases.

Mosquitoes were more abundant in the rice fields than in the forest environment (Table 2). The rice field environment provides large areas of aquatic habitat for mosquito breeding. The dynamics of culicids in the perimeter of the Kou valley where rice is grown are essentially influenced by two factors: the season and phase of rice cultivation (watering, heading, ripening stages). *Anopheles gambiae* larvae, for example, are mainly present during the watering phase of the rice paddies, then disappear during the rice growth phase due to shading by the rice and eutrophication of the water [41]. In the latter study, *An. gambiae* and, to a lesser extent, *An. funestus* were the main malaria vectors present in this rice-growing area [41], whereas in our study, only *An. gambiae* s.l. was collected. In general, *An. funestus* is not very abundant in savannah rice fields in West Africa [42]. However, in the present study the mosquito population was more diverse in the forest and rural environments (Fig. 2), and there was no statistically significant difference between the populations in these two environments. The high diversity of mosquito species compared with previous studies [30, 32] in these two types of environments can be explained by the maintenance of natural areas, despite anthropogenic pressure, including in the rural environment, and the productivity of natural breeding sites (tree hollows, sheathing leaves, rocks holes, etc.) which are suitable for many sylvatic mosquito species, particularly during the rainy season when rainfall is frequent and humidity high.

We found several mosquitoes species that are vectors of parasites (*Plasmodium* sp.) and/or arboviruses. Species of the genera *Culex, Aedes, Anopheles* and *Mansonia* are known to transmit pathogens [43, 44]. *Culex quinquefasciatus* and *Ae. aegypti*, which are both vectors of arboviruses in tropical regions, were abundant in our four environments. *Culex quinquefasciatus* is a vector for West Nile virus (WNV) and Usutu virus (USUV) [45–47]. A recent study on the seroprevalence of WNV and USUV in Ouagadougou and Bobo-Dioulasso, the two largest cities of Burkina Faso, showed the circulation of these arboviruses in donated blood [48]. The abundance of *Cx. quinquefasciatus* in different types of environment could constitute a potential risk favouring the emergence of WNV and USUV. Beside the potential for pathogen transmission, females of *Cx. quinquefasciatus* are very aggressive towards humans and constitute a night-time nuisance due to their biting, particularly in tropical urban environments [49]. *Aedes aegypti* is a known vector of several arboviruses of major public health importance, including dengue virus (DENV), yellow fever virus (YFV), chikungunya virus, and Zika virus (ZKV) [50–53]. YFV, DENV and ZKV circulate in Burkina Faso [37, 54, 55]. Despite the existence of a vaccine for YFV, an epidemic occurred in southeast Burkina Faso in 1983. The outbreak, which was rural, occurred in villages and herders’ camps located close to and in gallery forests, and *Ae. furcifer*, a sylvatic mosquito, was the main species involved in the transmission of the disease [56]. In 1983, 1984 and 1986, seven strains of YFV were isolated in wild mosquitoes in the region of Bobo-Dioulasso in remarkably similar circumstances [57]. The mosquitoes were from sylvatic areas, never from the towns, and were found at the end of the rainy season (in October and November). Only *Ae. luteocephalus* was found, a predominant potential vector of YFV in the region. These findings confirm that YFV regularly circulates in the southern savanna zone of West Africa, which therefore forms part of the endemic emergence zone. Cases of YFV still occur in the country, despite the routine Expanded Program of Immunization [58]. The risk of a new YFV outbreak is increasing due to the internal displacement of people as a consequence of terrorist conflicts, which may lead to a reduction in vaccine coverage and increase the risk of YFV emergence in several localities in Burkina Faso. Urban epidemics of YFV occurred in Abidjan, the capital of Côte d'Ivoire, during the armed unrest in the country in 2000 [59]. The circulation of DENV-2 in the sylvatic mosquito *Ae. luteocephalus* collected in the wooded savannah area of western Burkina Faso near Bobo-Dioulasso, was reported in 1980 [60], and DENV-2 was isolated from patients in 1982 in Ouagadougou city [61]. In 1986, two strains of DENV-2 were isolated from wild mosquitoes in the Bobo-Dioulasso region [57], one from *Ae. luteocephalus* in the sylvatic zone, the other from *Ae. aegypti* in the city centre. These findings indicated that these DENV-2 variants had distinct life cycles, one urban and the other sylvatic, and that the two may coexist in the same region. Other sylvatic *Aedes* species, such as *Ae. furcifer*, *Ae. vittatus*, *Ae. africanus* and *Ae. unilineatus,* have been associated with arbovirus transmission in the forest zone of Senegal [62, 63], confirming that the naturally humid areas of southern West Africa are a setting for enzootic circulation of dengue viruses. Since 2013, there have been several dengue outbreaks in the main cities of the country, Ouagadougou and Bobo-Dioulasso [54, 64]. The threat of YFV and DENV, as well as other viruses transmitted by *Aedes* which are present in the region, such as ZKV [55], could be increased by colonization by *Ae. albopictus*, a potential vector of these arboviruses, which are expanding rapidly in Africa and are already present in some of the neighbouring countries of Burkina Faso [39].

In Burkina Faso, the *An. gambiae* complex is composed of three species, *An. gambiae, An. arabiensis and An. coluzzii*, which are involved in the transmission of malaria parasites to humans in the country [65]. Previous studies reported that *An. arabiensis* was the main malaria vector in urban areas [31] and that *An. gambiae* and *An. coluzzii* were still predominant vectors in rural and peri-urban areas [66] in the western part of Burkina Faso. Other malaria vectors, such as *An. funestus* and *An. nili*, were observed in small numbers compared with *An. gambiae* s.l. These vectors may be more abundant in certain contexts in favourable ecological zones and constitute locally important vectors of species of* Plasmodium* that infect humans [67, 68]. *Anopheles stephensi*, an invasive urban Asian malaria vector, was not present in our samples. The recent establishment and expansion of *An. stephensi* in Africa suggest that it may become a serious threat to malaria control in urban areas of the continent [8].

## Conclusions

We identified 33 species of mosquitoes from four landscapes (urban, rural, rice fields and forest) in southwest Burkina Faso. *Culex quinquefasciatus*, *An. gambiae* s.l. and *Ae. aegypti* were the most abundant species in each environment. The species composition of the mosquito populations depended on the type of environment, with lower species diversity in highly human-modified environments such as urban areas and rice fields. The diversity and abundance of these mosquito vectors indicate that human populations in all of these environments may become more exposed to mosquito-borne diseases, in particular arboviruses, which are re-emerging or emerging in different regions of the world, including West Africa. Our main objective in the near future will be to screen the viromes (arboviruses and mosquito-specific viruses) associated with each species collected in this study to provide more information on mosquito vector-related risks in Burkina Faso.

## Data Availability

The datasets used during this article are fully available without restriction. Further inquiries can be directed to the corresponding authors.
